# Supplementary material for: Inhibition of polymorphonuclear cells averts cytotoxicity against hypoimmune cells in xenotransplantation
Source: Nat Commun. 2025 Apr 18;16:3706. doi: 10.1038/s41467-025-58774-7 (PMC12008267; doi:10.1038/s41467-025-58774-7)
Supplement: Supplementary file 1 — Supplementary Information [file 41467_2025_58774_MOESM1_ESM.pdf]

Inhibition of polymorphonuclear cells averts cytotoxicity against hypoimmune cells in xenotransplantation

Xiaomeng Hu[1,2], Grigol Tediashvili[1], Alessia Gravina[1], Jonathan Stoddard[3], Trevor J. McGill[3], Andrew J. Connolly[4], Tobias Deuse[1,7], Sonja Schrepfer[1,2,5,6,7]

[1] Department of Surgery, Division of Cardiothoracic Surgery, Transplant and Stem Cell Immunobiology (TSI)-Lab, University of California San Francisco, San Francisco, California, USA.
[2] Sana Biotechnology Inc., 1 Tower Place, South San Francisco, California, USA.
[3] Division of Neuroscience, Oregon National Primate Research Center, Oregon Health & Science University, Portland, Oregon, USA.
[4] Department of Pathology, University of California-San Francisco, San Francisco, California, USA.
[5] Department of Surgery, Cedars-Sinai Medical Center, Los Angeles, California, USA.
[6] Board of Governors Regenerative Medicine Institute, Cedars-Sinai Medical Center, Los Angeles, California, USA.
[7] Contributed equally.

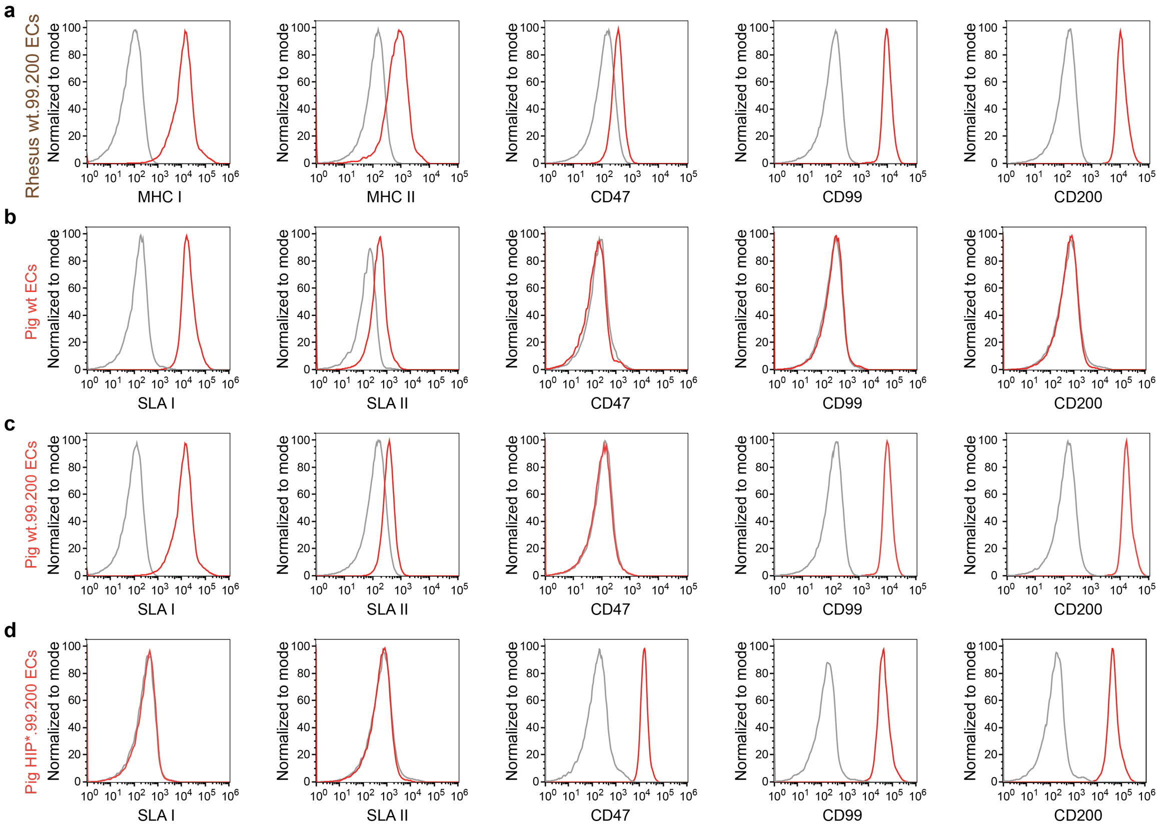

Supplementary Figure 1: ***Engineering of rhesus monkey and pig ECs.***
a Rhesus wt ECs were transduced to overexpress human CD99 and CD200. **b** Phenotypic characterization of pig wt ECs. **c** Pig wt ECs were transduced to overexpress human CD99 and CD200. **d** Pig HIP* ECs were depleted of SLA class I and II and overexpressed human CD47 and were additionally transduced to overexpress human CD99 and CD200. Representative flow cytometry histograms are shown of two independent analyses. The isotype controls are shown in grey.

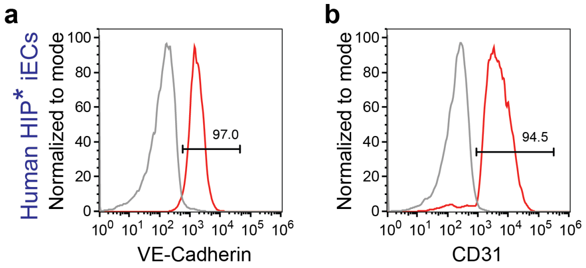

Supplementary Figure 2: ***Phenotyping of human HIP* iECs.***

a, b Human HIP* iPSCs were differentiated into iECs and their expression of VE-Cadherin (a) or CD31 (b) was assessed by flow cytometry (Representative histograms of two independent experiments). The isotype controls are shown in grey. The percentage of positive cells is presented.

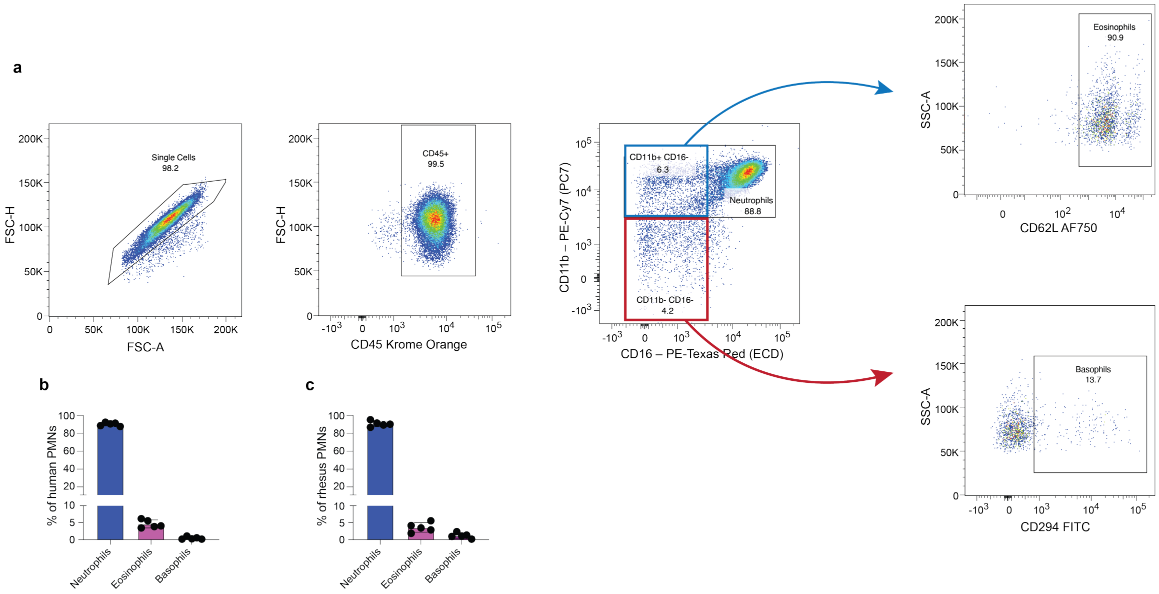

Supplementary Figure 3: ***Phenotyping of human and rhesus PMN subpopulations.*** **a** Human or rhesus PMNs were pre-gated on CD45+ cells and single cells. Granulocyte subpopulations were defined using expression patterns of CD11b, CD16, CD62L, and CD294. **b, c** Human (b) and rhesus (c) PMNs were mostly neutrophils (CD11b+, CD16+), with lower numbers of eosinophils (CD11b+, CD16-, CD62L+) and basophils (CD11b-, CD16-, CD294+). Five human donors and 5 rhesus donors were used (mean ± SD, n=5 donors, all individual data are shown).
