# Supplementary material for: Inhibition of polymorphonuclear cells averts cytotoxicity against hypoimmune cells in xenotransplantation
Source: Nat Commun. 2025 Apr 18;16:3706. doi: 10.1038/s41467-025-58774-7 (PMC12008267; doi:10.1038/s41467-025-58774-7)
Supplement: Supplementary file 2 — Reporting Summary [file 41467_2025_58774_MOESM2_ESM.pdf]

nature portfolio

Corresponding author(s): Sonja Schrepfer

Last updated by author(s): Mar 12, 2025

Reporting Summary

Nature Portfolio wishes to improve the reproducibility of the work that we publish. This form provides structure for consistency and transparency in reporting. For further information on Nature Portfolio policies, see our Editorial Policies and the Editorial Policy Checklist.

Statistics

For all statistical analyses, confirm that the following items are present in the figure legend, table legend, main text, or Methods section.

n/a	Confirmed	
☐	☒	The exact sample size (*n*) for each experimental group/condition, given as a discrete number and unit of measurement
☐	☒	A statement on whether measurements were taken from distinct samples or whether the same sample was measured repeatedly
☒	☐	The statistical test(s) used AND whether they are one- or two-sided<br>*Only common tests should be described solely by name; describe more complex techniques in the Methods section.*
☒	☐	A description of all covariates tested
☒	☐	A description of any assumptions or corrections, such as tests of normality and adjustment for multiple comparisons
☐	☒	A full description of the statistical parameters including central tendency (e.g. means) or other basic estimates (e.g. regression coefficient) AND variation (e.g. standard deviation) or associated estimates of uncertainty (e.g. confidence intervals)
☒	☐	For null hypothesis testing, the test statistic (e.g. *F*, *t*, *r*) with confidence intervals, effect sizes, degrees of freedom and *P* value noted<br>*Give P values as exact values whenever suitable.*
☒	☐	For Bayesian analysis, information on the choice of priors and Markov chain Monte Carlo settings
☒	☐	For hierarchical and complex designs, identification of the appropriate level for tests and full reporting of outcomes
☒	☐	Estimates of effect sizes (e.g. Cohen's *d*, Pearson's *r*), indicating how they were calculated

Our web collection on statistics for biologists contains articles on many of the points above.

Software and code

Policy information about availability of computer code

Data collection	FlowJo 10.8.1 was used to analyze flow cytometric data. Prism9 was used to make graphs. XCelligence assays were measured with the RTCA software.
Data analysis	Data were automatically analyzed in the RTCA software.

For manuscripts utilizing custom algorithms or software that are central to the research but not yet described in published literature, software must be made available to editors and reviewers. We strongly encourage code deposition in a community repository (e.g. GitHub). See the Nature Portfolio guidelines for submitting code & software for further information.

Data

Policy information about availability of data

All manuscripts must include a data availability statement. This statement should provide the following information, where applicable:

- Accession codes, unique identifiers, or web links for publicly available datasets
- A description of any restrictions on data availability
- For clinical datasets or third party data, please ensure that the statement adheres to our policy

All data generated or analysed during this study are included in this published article. No pre-established data exclusion method was used. No clinical data was included.

Research involving human participants, their data, or biological material

Policy information about studies with human participants or human data. See also policy information about sex, gender (identity/presentation), and sexual orientation and race, ethnicity and racism.

Reporting on sex and gender	N.A.
Reporting on race, ethnicity, or other socially relevant groupings	N.A.
Population characteristics	N.A.
Recruitment	N.A.
Ethics oversight	N.A.

Note that full information on the approval of the study protocol must also be provided in the manuscript.

Field-specific reporting

Please select the one below that is the best fit for your research. If you are not sure, read the appropriate sections before making your selection.

☒ Life sciences ☐ Behavioural & social sciences ☐ Ecological, evolutionary & environmental sciences

For a reference copy of the document with all sections, see nature.com/documents/nr-reporting-summary-flat.pdf

Life sciences study design

All studies must disclose on these points even when the disclosure is negative.

Sample size	We report on individual monkeys and every animal is plotted individually. Four monkeys per group seemed appropriate from a scientific, financial, and ethical standpoint.
Data exclusions	No pre-established data exclusion method was used. No data were excluded.
Replication	Every monkey is reported. No additional monkeys were used to replicate the findings.
Randomization	Animals were randomly assigned to experimental groups.
Blinding	The investigators were not blinded but the pathologist was blinded.

Reporting for specific materials, systems and methods

We require information from authors about some types of materials, experimental systems and methods used in many studies. Here, indicate whether each material, system or method listed is relevant to your study. If you are not sure if a list item applies to your research, read the appropriate section before selecting a response.

Materials & experimental systems

n/a	Involved in the study	
☐	☒	Antibodies
☐	☒	Eukaryotic cell lines
☒	☐	Palaeontology and archaeology
☐	☒	Animals and other organisms
☒	☐	Clinical data
☒	☐	Dual use research of concern
☒	☐	Plants

Methods

n/a	Involved in the study	
☒	☐	ChIP-seq
☐	☒	Flow cytometry
☒	☐	MRI-based neuroimaging

Antibodies

Antibodies used	Flow cytometry and cell sorting<br>To assess SLA class I expression, pig ECs were incubated with a AF647-conjugated anti-SLA-I antibody (clone JM1E3, cat.no. MCA2261A647, Bio-Rad, Hercules, CA) or AF647-conjugated IgG1 isotype-matched control antibody (cat.no. MCA928A647, Bio-Rad).

To assess SLA-II expression, cells were incubated with a PE-conjugated anti-SLA-II DR antibody (clone 2E9/13, cat.no. MCA2314GA, Bio-Rad) or PE-conjugated IgG2b isotype-matched control antibody (cat.no. MCA691PE, Bio-Rad).
For the detection of rhesus MHC-I and MHC-II on rhesus ECs, an APC-conjugated anti-HLA-A,B,C antibody (clone G46_2.6, cat.no. 555555, BD Biosciences) or APC-conjugated IgG1 isotype-matched control antibody (clone MOPC-21, cat.no. 554681, BD Biosciences) and an AF647-conjugated anti-HLA-DR,DP,DQ antibody (clone Tu39, cat.no. 563591, BD Biosciences) or AF647-conjugated IgG2a isotype-matched control antibody (clone G155–178, cat.no. 565357, BD Biosciences) were used, respectively.
To assess CD47 expression, a FITC-conjugated anti-CD47 antibody (clone CC2C6, cat.no. 323106, Biolegend) or FITC-conjugated IgG1 isotype-matched control antibody (clone MOPC-21, cat.no. 400107, Biolegend) was used.
For the detection of CD99, an APC-conjugated anti-CD99 antibody (clone hec2, cat.no. 398203, Biolegend) or APC-conjugated IgG1k isotype-matched control antibody (clone MOPC-21, cat.no. 400119, Biolegend) was used.
An APC-conjugated anti-human CD200 antibody (clone A18042B, cat.no. 399807, Biolegend) or APC-conjugated IgG1k isotype-matched control antibody (clone MOPC-21, cat.no. 400119, Biolegend) was used for the detection of CD200.
The endothelial cell phenotype of human HIP* iECs was assessed using a FITC-conjugated anti-VE-Cadherin antibody (CD144) (clone QA20A44, cat.no. 376509, Biolegend) or FITC-conjugated IgG1k isotype-matched control antibody (clone MOPC-21, cat.no. 400107, Biolegend) and an APC-conjugated anti-CD31 antibody (clone WM59, cat.no. 303115, Biolegend) or APC-conjugated IgG1k isotype-matched control antibody (clone MOPC-21, cat.no. 400119, Biolegend).
Rhesus NK cells were sorted using a FITC-conjugated anti-CD8 antibody (clone LT8, cat.no. ab28010, Abcam, 1:5) and a PE-conjugated anti-NKG2A antibody (clone REA110, cat.no. 130-114-092, Miltenyi, 1:50).
Rhesus T cells were sorted using an APC-conjugated anti-CD3 antibody (clone 10D12, cat.no. 130-123-790, Miltenyi, 1:50).
Ex vivo primed human or macaque T cells were sorted using an APC-conjugated mouse anti-human CD3 antibody (clone SP34-2, cat.no. 557597, BD Biosciences, concentration 0.01 mg/ml) together with the APC-conjugated IgG1κ isotype-matched control antibody (clone MOPC-21, cat.no. 550854, BD Biosciences, concentration 0.01 mg/ml) and a BV421-conjugated anti-human CD8 antibody (clone SK1, cat.no. 344748, BioLegend, concentration 0.005 mg/ml) together with the BV421-conjugated IgG1κ isotype-matched control antibody (clone MOPC-21, cat.no. 400157, BioLegend, concentration 0.005 mg/ml).

Immunohistochemistry
Primary antibodies were used as appropriate: Myeloperoxidase (MPO) (cat.no. PA5-16672, Invitrogen) and CD31 (cat.no. ab28364, Abcam).

Validation

Each antibody was tested with positive and negative control prior to staining the samples. Antibody concentration were gathered from vendors datasheet. Isotype and tested antibody were concentration matched.

Eukaryotic cell lines

Policy information about cell lines and Sex and Gender in Research

Cell line source(s)	Rhesus primary ECs were purchased from ATCC (CRL-1780, cat.no. RF/6A, ATCC, Manassas, VA). Rhesus primary PBMCs were purchased from HumanCells Biosciences (cat.no. M5-011, HumanCells, Milpitas, CA). Pig primary ECs were purchased from Cell Biologics (cat.no. P-6065, Cell Biologics, Chicago, IL).
Authentication	No authentication was performed
Mycoplasma contamination	All cells were tested for mycoplasma contamination.
Commonly misidentified lines (See ICLAC register)	No commonly misidentified cell lines were used.

Animals and other research organisms

Policy information about studies involving animals; ARRIVE guidelines recommended for reporting animal research, and Sex and Gender in Research

Laboratory animals	One female blood type AB rhesus monkey (Macaca mulatta, 2 years of age) was used at the Oregon national primate research center. Eight blood type A cynomolgus monkeys (Macaca fascicularis, 1-2 years of age, ~1-2 kg) were selected at the Alpha Genesis Primate Research Center. Five were female and 3 were male.
Wild animals	N.A.
Reporting on sex	The sex of the monkeys is reported.
Field-collected samples	N.A.
Ethics oversight	The Animal Welfare Act with protocols approved by the Institutional Animal Care and Use Committee at the Oregon Health and Science University and the Alpha Genesis Institutional Animal Care and Use Committee and regulated by the US Department of Agriculture were used.

Note that full information on the approval of the study protocol must also be provided in the manuscript.

Plants

Seed stocks | N.A.

Novel plant genotypes | N.A.

Authentication | N.A.

Flow Cytometry

Plots

Confirm that:

☒ The axis labels state the marker and fluorochrome used (e.g. CD4-FITC).

☒ The axis scales are clearly visible. Include numbers along axes only for bottom left plot of group (a 'group' is an analysis of identical markers).

☒ All plots are contour plots with outliers or pseudocolor plots.

☒ A numerical value for number of cells or percentage (with statistics) is provided.

Methodology

Sample preparation	Rhesus PBMCs were isolated from fresh blood by Ficoll separation and NK and T cells were sorted.
Instrument	FACSAria Fusion
Software	The FlowJo 10.8.1 software was used.
Cell population abundance	For flow cytometry analysis, more than 10,000 positive cells were measured. Cell sorting was gated for the desired population and sorted for the cell amount needed for assays.
Gating strategy	Samples were gated in FSC/SSC for the correct cell size and live cells. Isotype was measured for each sample as defined as unspecific staining threshold.

☒ Tick this box to confirm that a figure exemplifying the gating strategy is provided in the Supplementary Information.
